# The association between childhood neighborhood relationship and mental health in middle and later life: Evidence from the China Health and Retirement Longitudinal Study

**DOI:** 10.1186/s13690-021-00714-0

**Published:** 2021-11-22

**Authors:** Jian Sun, Hongye Luo, Chaofan Li, Qianqiang Wang

**Affiliations:** 1 School of International and Public Affairs, Shanghai Jiao Tong University, Shanghai, China; 2 Saw Swee Hock School of Public Health, National University of Singapore, Singapore, Singapore; 3 School of Information and Management, Guangxi Medical University, Guangxi Zhuang Autonomous Region, Nanning, China; 4 Centre for Health Management and Policy Research, School of Public Health, Cheeloo College of Medicine, Shandong University, Jinan, Shandong China; 5 NHC Key Lab of Health Economics and Policy Research (Shandong University), Jinan, Shandong China; 6 School of Humanities and Social Science, Guangxi Medical University, Guangxi Zhuang Autonomous Region, Nanning, China

**Keywords:** Childhood neighborhood relationship, Mental health, Middle-aged and older adults, China, CHARLS

## Abstract

**Background:**

It is unclear that whether childhood neighborhood relationship is associated with mental health among middle-aged and older adults. To overcome this research gap, this study aimed to investigate the association between childhood neighborhood relationship and mental health among the middle-aged and older adults in China.

**Methods:**

The data of this study was sourced from the 2014 and 2015 waves of China Health and Retirement Longitudinal Study. We used ordinary least squares and logit regression models to explore the association between childhood neighborhood relationship and mental health among the middle-aged and older adults in China.

**Results:**

The regression results indicate that the middle-aged and older adults who lived in place where neighbors had close-knit relationships at childhood was significantly associated with decreased odds of suffering from depressive symptoms (OR = 0.4259, *p* < 0.001). Furthermore, compared to the middle-aged and older adults who lived in place where neighbors were not close-knit at childhood, those who lived in place where neighbors were close-knit at childhood had a reduced CES–D score (coefficient = − 2.7822, *p* < 0.001).

**Conclusion:**

This study demonstrates the importance of living in place where neighbors had close-knit relationships at childhood. The integrated interventions, including maintaining close-knit neighborhood relationships and strengthening the construction of community, may be useful to improve mental health.

## Background

Mental health affects the lives of people. Specifically speaking, people with mental disorders are more likely to have a lower quality of life. As the physiological functions gradually decline with age, middle-aged and older adults have high incidence of dementia, depression, and other mental diseases [1]. Some studies reveal that the mental health of the middle-aged and older adults is poor. Lei et al. used the data which was obtained from the 2011/2012 wave of China Health and Retirement Longitudinal Study and found that 30% of men and 43% of women who aged 45 years and older suffered from depressive symptoms [2]. In addition, the World Health Organization reports that approximately 15% of adults who aged 60 years and older suffer from a mental disorder [3].

Several studies have explored the association between neighborhood and mental health. Curry et al. reported that neighborhood violence is significantly associated with depressive symptoms [4]. Furthermore, a study which was conducted by Perez et al. reveals that people with greater neighborhood social cohesion is significantly related to fewer depressive symptoms [5]. Moreover, Miao et al. used the data which was obtained from the first wave of Shanghai Urban Neighborhood Survey (SUNS) and reported that Chinese older adults that lived in neighborhoods of lower socioeconomic status are more likely to interact with their neighbors and thus perceive a higher level of social cohesion, and social cohesion, in turn, is significantly linked to a lower rate of depression [6]. Araya et al. found that the perceived quality of the neighbourhood is significantly associated with mental health [7].

Overall, previous studies have confirmed the association between neighborhood and mental health. Furthermore, life course theory emphasizes analyzing the association between experience of people and health outcome from the perspective of life course [8], and childhood experience, such as living in place where neighbors had close-knit relationships at childhood, is significantly correlated with health outcome in middle and later life [9]. Therefore, we hypothesize that childhood neighborhood relationship is significantly associated with mental health in middle and later life. However, we find that previous studies do not explore the association between childhood neighborhood relationship and mental health among the middle-aged and older adults. To overcome this research gap, this study used the data which was obtained from the China Health and Retirement Longitudinal Study (CHARLS) and aimed to investigate the association between childhood neighborhood relationship and mental health among the Chinese middle-aged and older adults. The results of this study can provide a deeper understanding on the association between childhood neighborhood relationship and mental health among the middle-aged and older adults in China, which is of great significance to improve their health outcome.

## Methods

### Data source

The data of this study was sourced from the 2014 and 2015 waves of China Health and Retirement Longitudinal Study (CHARLS). We got the CHARLS data through its official website (http://charls.pku.edu.cn/). The CHARLS is a nationally representative longitudinal survey of persons in China who aged 45 years and older and their spouses [10]. Considering the fact that the CHARLS contains rich information about middle-aged and older adults’ childhood neighborhood relationship, mental health, demographic characteristics, socioeconomic status, and lifestyle, we employed it explore the association between childhood neighborhood relationship and mental health among the middle-aged and older adults in China. The 2014 wave of CHARLS collected the data concerning life history, which includes residency history, education history, health history, health care history, childhood history, wealth history, and so on. Furthermore, the 2015 wave of CHARLS collected the data concerning demographic characteristics, family information, health status and functioning, health care and insurance, socioeconomic status, lifestyle, and so on. Because the 2015 wave of CHALRS does not have the data of childhood neighborhood relationship, while the 2014 wave of CHARLS has it, we matched the participants from the 2014 and 2015 waves of CHARLS according to the individual ID. After data cleaning, a total of 5293 participants who aged 45 years and older were included in this study.

### Variables

#### Dependent variables

**Fig. 1:**
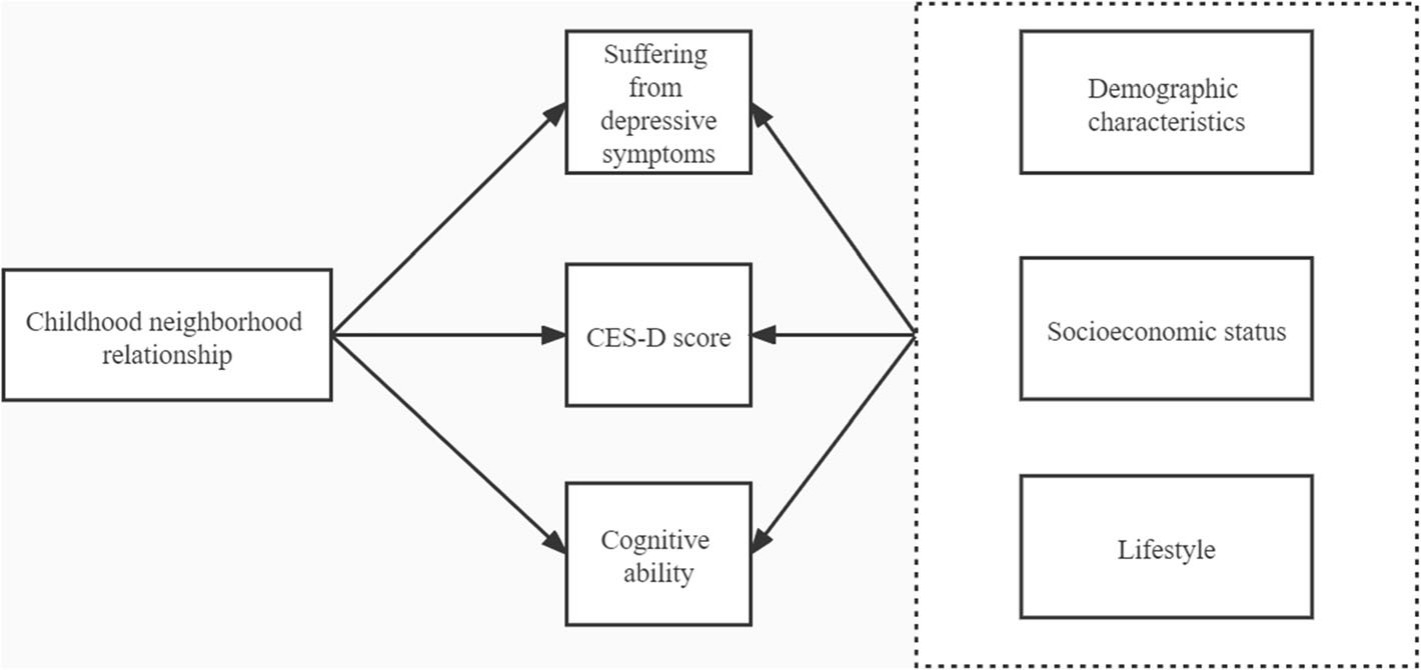
Relationship of the variables used in this study

Figure 1 shows the relationship of the variables used in this study. In this study, mental health is the dependent variable. Mental health was measured by depression status and cognitive ability. We used the 10-item Center for Epidemiological Studies–Depression Scale (CES–D 10), which has been widely used in previous studies [11–16]. This scale focuses on the depressed mood of people. Previous studies have confirmed the reliability and validity of this scale [17, 18]. This scale has ten questions. Among the ten questions, eight of which were negatively-oriented questions, such as “I was bothered by things that don’t usually bother me”, “I had trouble keeping my mind on what I was doing”, “I felt depressed”, “I felt everything I did was an effort”, and “I felt fearful”; two of which were positively-oriented questions, including “I felt hopeful about the future” and “I was happy”. All the ten questions from the CHARLS have four response levels, which range from rarely or none of the time, some or a little of the time, occasionally or a moderate amount of the time, to most or all of the time. In this study, the two positively-oriented questions were measured by a 4-point Likert scale. Furthermore, we reverse-coded the negatively-oriented questions, and they were measured by a 4-point Likert scale. We summed the ten questions to get a total CES–D score for each adult. The CES–D score was used to analyse depression status, which ranges from 0 to 30 and a higher value suggesting a higher degree of depressed mood. Moreover, whether the middle-aged and older adults suffered from depressive symptoms was employed to analyse depression status. Following Andresen et al. [19], a CES–D score of 10 and over was identified as suffering from depressive symptoms.

Following Cui et al. [20], cognitive ability was measured by several cognitive tasks, which capture six dimensions of the middle-aged and older adults’ cognitive abilities (short-term memory, reaction, calculation, drawing, naming, and language), including subtracting 7 from 100 for 5 times, naming today’s date (including month, day, year, and season), recalling the day of week, and redrawing a picture which was shown by the interviewer. The score of cognitive ability ranges from 0 to 11, with a higher score indicating a better cognitive status.

#### Exposure variable

In this study, childhood neighborhood relationship is the exposure variable. Childhood neighborhood relationship is a dummy variable, which indicates whether the middle-aged and older adults lived as a child in place where neighbors had close-knit relationships or not.

#### Control variables

Three types of control variables were selected in this study. The control variables were collected in 2015. The first type of control variables described the demographic characteristics, which include age, gender, marital status, and residency area. The second type of control variables described the socioeconomic status, which include education status, household income. The third type of control variables described the lifestyle, including drinking, sleeping time, and social interaction. In addition, a set of dummies for provinces were controlled in order to rule out the region fixed effects.

This study conducted a variance inflation factor (VIF) test, with test results suggesting that the mean VIF value was 1.75 and all the VIF values for variables which were used in this study were much lower than the critical value of 10, which indicates that there was no serious multicollinearity across the regression models.

### Statistical analyses

In this study, we used a dummy variable to indicate whether the middle-aged and older adults suffered from depressive symptoms or not. Based on previous studies [16, 21, 22], logit regression models were used to analyse the association between childhood neighborhood relationship and odds of suffering from depressive symptoms. In order to reduce the potential effect of heteroscedasticity, we reported robust standard errors that clustered at the community level. The econometric model for the association between childhood neighborhood relationship and the odds of suffering from depressive symptoms can be written as:

log ($$ \frac{p_i}{1-{p}_i} $$) = *α*_0_ *+ α*_1_ * *CNR*_*i*_ *+* $$ \sum \limits_{j=1}^n{a}_j\ast {X}_{ji} $$  *+ δ*_*s*_ *+ ε*_*i*_ (1).

where *p*_*i*_ indicates the possibility of suffering from depressive symptoms for the adult *i*, *p*_*i*_ / (1 - *p*_*i*_) represents the odds of suffering from depressive symptoms, *CNR*_*i*_ denotes childhood neighborhood relationship, *X*_*ji*_ suggests the control variables, *α*_0_ is the intercept term, *α*_1_ stands for the coefficient of childhood neighborhood relationship, which is our main interest, *α*_*j*_ indicates coefficients of the control variables, *δ*_*s*_ suggests the province fixed effects, and *ε*_*i*_ is the error term.

Given the fact that CES–D score and cognitive ability were continuous variables, we used ordinary least squares (OLS) regression models for statistical analysis. The econometric model for the association between childhood neighborhood relationship and CES–D score can be written as:

*CES–D score*_*i*_ = *β*_0_ *+ β*_1_ * *CNR*_*i*_ *+* $\sum \limits_{j=1}^n{\beta}_j\ast {X}_{ji}$  *+ δ*_*s*_ *+ ε*_*i*_ (2).

where *CNR*_*i*_ denotes childhood neighborhood relationship, *X*_*ji*_ represents the control variables, *β*_0_ stands for the intercept term, *β*_1_ is the coefficient of childhood neighborhood relationship, which is our main interest, *β*_*j*_ indicates the coefficients of the control variables, *δ*_*s*_ suggests the province fixed effects, and *ε*_*i*_ is the error term.

The econometric model for the association between childhood neighborhood relationship and cognitive ability can be written as:

*Cognitive ability*_*i*_ = *γ*_0_ *+ γ*_1_ * *CNR*_*i*_ *+* ${\sum}_{j=1}^n{\gamma}_j\ast {X}_{ji}$  *+ δ*_*s*_ *+ ε*_*i*_ (3).

where *CNR*_*i*_ denotes childhood neighborhood relationship, *X*_*ji*_ represents the control variables, *γ*_0_ indicates the intercept term, *γ*_1_ is the coefficient of childhood neighborhood relationship, which is our main interest, *γ*_*j*_ indicates the coefficients of the control variables, *δ*_*s*_ suggests the province fixed effects, and *ε*_*i*_ is the error term.

Given the fact that propensity score matching (PSM) can address the self-selection bias which is caused by observable characteristics [23, 24], we used it to conduct a robustness check. PSM which is based on Neyman-Rubin-Holland counterfactual framework (potential outcome framework), was put forward by Rosenbaum and Rubin [25]. In addition, PSM is a non-parametric estimation method [26], which makes the estimation results independent of the function of model [27]. However, PSM depends on large sample size and the matching results between treatment and control groups [28]. The propensity score reflects the probability of receiving treatment for the adult [29]. The process of PSM includes two stages. The first stage is to estimate the propensity score using logit or probit regression model, and the second stage is to conduct sample matching and estimate treatment effect [30]. In this study, we used a logit regression model to estimate the propensity score. The logit regression model used for estimating propensity score is as follows:

*P*(*X*_*i*_) = *Pr*{*D*_*i*_ = 1 | *X*_*i*_} = $$ \frac{\exp \left(\beta Xi\right)}{1+\exp \left(\beta Xi\right)} $$ + *ε*_*i*_ (4).

Average treatment effect on the treated (ATT) is estimated as follows:

*ATT* = *E*(*Y* ^*T*^ *- Y* ^*C*^ | *D*_*i*_ = 1) = *E*(*Y* ^*T*^ | *D*_*i*_ = 1) - *E*(*Y* ^*C*^ | *D*_*i*_ = 1) (5).

where *E*(*Y* ^*T*^ | *D*_*i*_ = 1) indicates the average mental health of the middle-aged and older adults who lived as a child in place where neighbors had close-knit relationships, and *E*(*Y* ^*C*^ | *D*_*i*_ = 1) stands for the counterfactual outcome, which indicates what the average mental health of the middle-aged and older adults who lived as a child in place where neighbors had close-knit relationships would have been if they had lived as a child in place where neighbors did not have close-knit relationship.

Doubly robust estimation is a non-parametric estimation method. Given the fact that doubly robust estimation combines the advantage of propensity score weighting and regression model and can report robust estimation results [26, 31], we used it to conduct a robustness check.

## Results

### Characteristics of the study population

Table 1 presents the characteristics of the study population. About 50% of the participants aged 60 years and older, and 58% of the participants were male. More than 90% of the participants were married, and approximately 82% of the participants lived in rural areas. Moreover, approximately 93% of the participants were literate, and the mean value of household income was RMB 12,276.54 (US$ 1766.59). In addition, more than 41% of the participants drank alcohol. The mean value of sleeping time for the participants was 6.46 h every day, and about 60% of the participants took part in social interaction.

**Table 1:** Characteristics of the study population

Variable	All (*N* = 5293)
Age	
45–59, n (%)	2650 (50.07)
≥ 60, n (%)	2643 (49.93)
Gender	
Female^a^, n (%)	2223 (42.00)
Male, n (%)	3070 (58.00)
Marital status	
Single, divorced or widowed^a^, n (%)	503 (9.50)
Married, n (%)	4790 (90.50)
Residency area	
Rural areas^a^, n (%)	4362 (82.41)
Urban areas, n (%)	931 (17.59)
Education status	
Illiterate^a^, n (%)	381 (7.20)
Literate, n (%)	4912 (92.80)
Household income (RMB)	
Mean (SD)	12,276.54 (85,825.17)
Drinking	
No^a^, n (%)	3114 (58.83)
Yes, n (%)	2179 (41.17)
Sleeping time (Hour)	
Mean (SD)	6.46 (1.69)
Social interaction	
No^a^, n (%)	2104 (39.75)
Yes, n (%)	3189 (60.25)

^a^ indicates the reference group

### Association between childhood neighborhood relationship and odds of suffering from depressive symptoms

Table 2 displays the logit regression results of the association between childhood neighborhood relationship and odds of suffering from depressive symptoms among the middle-aged and older adults. In order to test the stability of the regression models, we used the stepwise regression methods to incorporate variables into the regression models in turn. Model 1 indicates that the middle-aged and older adults who lived in place where neighbors had close-knit relationships at childhood was significantly associated with decreased odds of suffering from depressive symptoms by 55.92% (OR = 0.4408, *p* < 0.001). Furthermore, when the demographic characteristics variables were controlled in Model 2, the odds ratio of childhood neighborhood relationship slightly decreased from 0.4408 to 0.4333, and the significance level remained unchanged. Moreover, when the demographic characteristics variables and socioeconomic status variables were added in Model 3, the odds ratio of childhood neighborhood relationship slightly increased from 0.4333 to 0.4378, while the significance level remained unchanged. In addition, when the lifestyle variables were further controlled in Model 4, the odds ratio of childhood neighborhood relationship slightly decreased from 0.4378 to 0.4259, and the significance level remained unchanged. This means that living in place where neighbors had close-knit relationships at childhood was significantly related to reduced odds of suffering from depressive symptoms by 57.41% after adjusting for the control variables.

**Table 2:** Logit regression results of the association between childhood neighborhood relationship and odds of suffering from depressive symptoms among the middle-aged and older adults

Variable	Model 1	Model 2	Model 3	Model 4
Childhood neighborhood relationship	0.4408^***^	0.4333^***^	0.4378^***^	0.4259^***^
	(0.0657)	(0.0654)	(0.0667)	(0.0697)
	[0.3292,0.5903]	[0.3223,0.5824]	[0.3248,0.5901]	[0.3090,0.5870]
Age		1.0075	1.0054	1.0004
		(0.0043)	(0.0045)	(0.0045)
		[0.9992,1.0160]	[0.9967,1.0142]	[0.9915,1.0093]
Gender		0.5698^***^	0.5945^***^	0.6623^***^
		(0.0358)	(0.0391)	(0.0509)
		[0.5038,0.6446]	[0.5227,0.6763]	[0.5697,0.7699]
Marital status		0.6131^***^	0.6019^***^	0.6046^***^
		(0.0663)	(0.0651)	(0.0680)
		[0.4961,0.7578]	[0.4869,0.7441]	[0.4850,0.7538]
Residency area		0.5223^***^	0.5232^***^	0.5199^***^
		(0.0559)	(0.0561)	(0.0550)
		[0.4234,0.6443]	[0.4241,0.6456]	[0.4225,0.6397]
Education status			0.8146	0.8463
			(0.0928)	(0.0980)
			[0.6515,1.0185]	[0.6744,1.0619]
Household income			0.9880	0.9902
			(0.0081)	(0.0082)
			[0.9723,1.0040]	[0.9742,1.0065]
Drinking				0.9038
				(0.0710)
				[0.7749,1.0542]
Sleeping time				0.7765^***^
				(0.0172)
				[0.7436,0.8109]
Social interaction				0.7732^***^
				(0.0545)
				[0.6734,0.8878]
Constant	0.8203	1.2293	1.7312	12.9485^***^
	(0.2042)	(0.5041)	(0.7666)	(6.2420)
	[0.5036,1.3361]	[0.5503,2.7460]	[0.7268,4.1236]	[5.0337,33.3084]
Province fixed effects	Yes	Yes	Yes	Yes
Number of observations	5293	5293	5293	5293
Wald chi2	169.15^***^	386.02^***^	364.88^***^	464.35^***^
Pseudo R-squared	0.0199	0.0430	0.0439	0.0738

Robust standard errors that clustered at the community level are reported in parentheses. 95% confidence intervals are reported in square brackets. ^***^p < 0.001, ^**^p < 0.01, ^*^p < 0.05

### Association between childhood neighborhood relationship and CES–D score

Table 3 shows the OLS regression results of the association between childhood neighborhood relationship and CES–D score among the middle-aged and older adults. According to Model 1, we find that the middle-aged and older adults who lived in place where neighbors were close-knit at childhood had a significantly lower CES–D score than those who did not (coefficient = − 2.8597, *p* < 0.001). When we added the demographic characteristics variables to Model 2, the coefficient of childhood neighborhood relationship slightly increased from − 2.8597 to − 2.8145, while the regression significance level remained unchanged (Model 2). When we further added the socioeconomic status variables to Model 3, the OLS regression results show that the coefficient of childhood neighborhood relationship slightly increased from − 2.8145 to − 2.7917. When we further added the lifestyle variables to Model 4, the OLS regression results indicate that the coefficient of childhood neighborhood relationship slightly increased from − 2.7917 to − 2.7822. Furthermore, the association between childhood neighborhood relationship and CES–D score remained the same in terms of regression significance level (Model 4). In addition, the OLS regression results imply that compared to the middle-aged and older adults who lived in place where neighbors were not close-knit at childhood, those who lived in place where neighbors were close-knit at childhood had a reduced CES–D score by 2.7822 after adjusting for the control variables (Model 4).

**Table 3:** OLS regression results of the association between childhood neighborhood relationship and CES–D score among the middle-aged and older adults

Variable	Model 1	Model 2	Model 3	Model 4
Childhood neighborhood relationship	−2.8597^***^	−2.8145^***^	−2.7917^***^	−2.7822^***^
	(0.4596)	(0.4421)	(0.4430)	(0.4474)
	[−3.7630,-1.9564]	[−3.6834,-1.9456]	[−3.6624,-1.9210]	[−3.6616,-1.9029]
Age		0.0294^**^	0.0256^*^	0.0126
		(0.0099)	(0.0102)	(0.0099)
		[0.0100,0.0487]	[0.0055,0.0458]	[−0.0067,0.0320]
Gender		−1.5169^***^	−1.4417^***^	−1.1358^***^
		(0.1598)	(0.1695)	(0.1813)
		[−1.8310,-1.2027]	[−1.7749,-1.1085]	[−1.4922,-0.7795]
Marital status		−1.5151^***^	− 1.5388^***^	− 1.4544^***^
		(0.2958)	(0.2955)	(0.2937)
		[−2.0965,-0.9337]	[−2.1195,-0.9581]	[−2.0317,-0.8771]
Residency area		−1.5804^***^	−1.5683^***^	− 1.5787^***^
		(0.2270)	(0.2263)	(0.2217)
		[−2.0266,-1.1342]	[−2.0130,-1.1235]	[− 2.0145,-1.1430]
Education status			−0.4777	−0.3401
			(0.3224)	(0.3089)
			[−1.1115,0.1560]	[−0.9472,0.2671]
Household income			−0.0169	−0.0119
			(0.0183)	(0.0176)
			[−0.0529,0.0192]	[−0.0464,0.0227]
Drinking				−0.2301
				(0.1658)
				[−0.5559,0.0956]
Sleeping time				−0.7354^***^
				(0.0519)
				[−0.8374,-0.6334]
Social interaction				−0.7027^***^
				(0.1611)
				[−1.0193,-0.3861]
Constant	10.0259^***^	10.7144^***^	11.3856^***^	17.0428^***^
	(0.7760)	(1.0714)	(1.1400)	(1.1800)
	[8.5008,11.5511]	[8.6088,12.8201]	[9.1451,13.6260]	[14.7236,19.3620]
Province fixed effects	Yes	Yes	Yes	Yes
Number of observations	5293	5293	5293	5293
F statistics	15.40^***^	32.63^***^	32.81^***^	45.60^***^
R-squared	0.0356	0.0700	0.0706	0.1210

Robust standard errors that clustered at the community level are reported in parentheses. 95% confidence intervals are reported in square brackets. ^***^p < 0.001, ^**^p < 0.01, ^*^p < 0.05

### Association between childhood neighborhood relationship and cognitive ability

Table 4 presents the OLS regression results of the association between childhood neighborhood relationship and cognitive ability among the middle-aged and older adults. Model 1 reveals that childhood neighborhood relationship was not significantly linked to cognitive ability (coefficient = 0.3005, *p* > 0.05). When we added the demographic characteristics variables to Model 2, it is observed that the coefficient of childhood neighborhood relationship decreased from 0.3005 to 0.2662, while the regression significance level remained the same (Model 2). When the socioeconomic status variables were further added to Model 3, the OLS regression results indicate that the coefficient of childhood neighborhood relationship slightly decreased from 0.2662 to 0.2410 and the regression significance level remained unchanged. When we further controlled the lifestyle variables to Model 4, the OLS regression results reveal that the coefficient of childhood neighborhood relationship slightly decreased from 0.2410 to 0.2284. Moreover, the association between childhood neighborhood relationship and cognitive ability remained the same in terms of regression significance level (Model 4).

**Table 4:** OLS regression results of the association between childhood neighborhood relationship and cognitive ability among the middle-aged and older adults

Variable	Model 1	Model 2	Model 3	Model 4
Childhood neighborhood relationship	0.3005	0.2662	0.2410	0.2284
	(0.1547)	(0.1538)	(0.1531)	(0.1528)
	[−0.0035,0.6045]	[−0.0362,0.5685]	[− 0.0599,0.5419]	[− 0.0719,0.5287]
Age		−0.0155^***^	− 0.0117^***^	− 0.0104^**^
		(0.0033)	(0.0034)	(0.0035)
		[−0.0220,-0.0090]	[−0.0184,-0.0049]	[− 0.0172,-0.0036]
Gender		0.3627^***^	0.2847^***^	0.2584^***^
		(0.0600)	(0.0608)	(0.0672)
		[0.2447,0.4806]	[0.1651,0.4042]	[0.1262,0.3905]
Marital status		0.1874	0.2067^*^	0.2072^*^
		(0.0959)	(0.0959)	(0.0959)
		[−0.0011,0.3759]	[0.0182,0.3951]	[0.0188,0.3957]
Residency area		0.4832^***^	0.4636^***^	0.4446^***^
		(0.0760)	(0.0760)	(0.0765)
		[0.3339,0.6326]	[0.3142,0.6129]	[0.2942,0.5950]
Education status			0.5789^***^	0.5654^***^
			(0.1106)	(0.1102)
			[0.3616,0.7963]	[0.3488,0.7820]
Household income			0.0138^*^	0.0123^*^
			(0.0061)	(0.0061)
			[0.0018,0.0257]	[0.0003,0.0243]
Drinking				0.0401
				(0.0576)
				[−0.0731,0.1533]
Sleeping time				0.0262
				(0.0144)
				[−0.0022,0.0545]
Social interaction				0.1692^**^
				(0.0514)
				[0.0681,0.2703]
Constant	9.0330^***^	9.5566^***^	8.8187^***^	8.4998^***^
	(0.2155)	(0.3218)	(0.3534)	(0.3750)
	[8.6095,9.4565]	[8.9242,10.1889]	[8.1241,9.5134]	[7.7629,9.2367]
Province fixed effects	Yes	Yes	Yes	Yes
Number of observations	5293	5293	5293	5293
F statistics	6.46^***^	8.86^***^	9.18^***^	9.68^***^
R-squared	0.0179	0.0379	0.0447	0.0471

Robust standard errors that clustered at the community level are reported in parentheses. 95% confidence intervals are reported in square brackets. ^***^p < 0.001, ^**^p < 0.01, ^*^p < 0.05

### Robustness checks

In this section, we employed PSM and doubly robust estimations to carry out robustness checks on the previous regression results.

Firstly, we used k-nearest neighbor matching, radius matching, and nearest-neighbor matching within caliper from PSM to conduct a robustness check. PSM should satisfy the conditional independence assumption (CIA). Table 5 reports the PSM estimation results for the association between childhood neighborhood relationship and mental health among the middle-aged and older adults. The results of all the three estimation methods indicate that childhood neighborhood relationship was significantly associated with the possibility of suffering from depressive symptoms (*p* < 0.001). Furthermore, the estimation results also reveal that childhood neighborhood relationship was significantly linked to the CES–D score (*p* < 0.001). Moreover, it is found that childhood neighborhood relationship was not significantly related to cognitive ability (*p* > 0.05).

**Table 5:** PSM estimation results for the association between childhood neighborhood relationship and mental health among the middle-aged and older adults

	Depressive symptoms	CES–D score	Cognitive ability
K-nearest neighbor matching	−0.1697^***^	−2.5575^***^	0.2603
	(0.0416)	(0.5482)	(0.1640)
Number of observations	5172	5172	5172
Radius matching	−0.1675^***^	− 2.5220^***^	0.3054
	(0.0405)	(0.5343)	(0.1599)
Number of observations	5158	5158	5158
Nearest-neighbor matching within caliper	−0.1703^***^	−2.5645^***^	0.2650
	(0.0417)	(0.5494)	(0.1644)
Number of observations	5158	5158	5158

Standard errors are reported in parentheses. ^***^p < 0.001, ^**^p < 0.01, ^*^p < 0.05

Secondly, we employed augmented inverse-probability weighting (AIPW) and inverse-probability-weighted regression adjustment (IPWRA) from doubly robust estimation to perform a robustness check. Table 6 provides the doubly robust estimation results for the association between childhood neighborhood relationship and mental health among the middle-aged and older adults. The results of the two estimation methods from doubly robust estimation were similar to the regression results in the previous sections.

**Table 6:** Doubly robust estimation results for the association between childhood neighborhood relationship and mental health among the middle-aged and older adults

	Depressive symptoms		CES–D score		Cognitive ability	
	Model 1	Model 2	Model 3	Model 4	Model 5	Model 6
	AIPW	IPWRA	AIPW	IPWRA	AIPW	IPWRA
ATE	−0.1540^***^	− 0.1564^***^	−2.2830^***^	−2.3102^***^	0.2276	0.2191
	(0.0387)	(0.0388)	(0.4452)	(0.4485)	(0.1545)	(0.1516)
POmean	0.3977^***^	0.4001^***^	8.8157^***^	8.8430^***^	8.9263^***^	8.9348^***^
	(0.0383)	(0.0384)	(0.4396)	(0.4428)	(0.1525)	(0.1495)
ATT		−0.1558^***^		−2.2925^***^		0.2177
		(0.0390)		(0.4499)		(0.1523)
POmean		0.3993^***^		8.8224^***^		8.9388^***^
		(0.0386)		(0.4443)		(0.1503)
Number of observations	5293	5293	5293	5293	5293	5293

Robust standard errors are reported in parentheses. ^***^p < 0.001, ^**^p < 0.01, ^*^p < 0.05

To sum up, the results of PSM and doubly robust estimation reveal that our regression results in the previous sections were highly robust for further research.

### Heterogeneity analysis

To explore whether the association between childhood neighborhood relationship and mental health differs by age, gender, residency area, education status, and income, we incorporated the interaction terms into the regression models.

Table 7 displays the heterogeneity of the association between childhood neighborhood relationship and mental health among the middle-aged and older adults. We divided the sample into two age groups, the middle-aged adults (aged 45–59 years) and the older adults (aged 60 years and older). The results of heterogeneity analysis indicate that there was a statistically significant interaction term between age and childhood neighborhood relationship for the odds of suffering from depressive symptoms (OR = 0.4182, *p* < 0.05), indicating that age moderates the association between childhood neighborhood relationship and the odds of suffering from depressive symptoms. The sign of the interaction term means that childhood neighborhood relationship was significantly linked to a greater decrease in the odds of suffering from depressive symptoms for the older adults who aged 60 years and older than for the middle-aged adults who aged 45–59 years. In addition, it is worth noting that the interaction term between childhood neighborhood relationship and age for CES–D score was statistically significant (coefficient = − 3.0119, *p* < 0.001), which indicates that age moderates the association between childhood neighborhood relationship and CES–D score. This reveals that childhood neighborhood relationship was significantly associated with a greater decrease in CES–D score for the older adults who aged 60 years and older than for the middle-aged adults who aged 45–59 years.

**Table 7:** Heterogeneity of the association between childhood neighborhood relationship and mental health among the middle-aged and older adults

	Depressive symptoms	CES–D score	Cognitive ability
**A. Age difference**			
Childhood neighborhood relationship	0.7154	− 0.9965	0.2927
	(0.1936)	(0.5703)	(0.2362)
	[0.4209,1.2157]	[− 2.1175,0.1244]	[− 0.1714,0.7569]
Age60	2.8686^**^	3.2139^***^	0.2314
	(1.0546)	(0.8873)	(0.3130)
	[1.3955,5.8965]	[1.4701,4.9577]	[−0.3838,0.8466]
Childhood neighborhood relationship * age60	0.4182^*^	−3.0119^***^	−0.1170
	(0.1475)	(0.8514)	(0.3128)
	[0.2095,0.8348]	[−4.6853,-1.3386]	[−0.7317,0.4977]
**B. Gender difference**			
Childhood neighborhood relationship	0.5233^*^	−2.5033^**^	0.3867
	(0.1433)	(0.8090)	(0.2618)
	[0.3059,0.8952]	[−4.0933,-0.9133]	[−0.1279,0.9013]
Gender	0.8914	−0.7374	0.4845
	(0.3127)	(1.0231)	(0.3118)
	[0.4482,1.7729]	[−2.7482,1.2734]	[−0.1283,1.0972]
Childhood neighborhood relationship * gender	0.7348	−0.4097	−0.2325
	(0.2617)	(1.0039)	(0.3129)
	[0.3657,1.4768]	[−2.3827,1.5633]	[−0.8474,0.3824]
**C. Residency area difference**			
Childhood neighborhood relationship	0.4147^***^	−2.9815^***^	0.1919
	(0.0701)	(0.4508)	(0.1691)
	[0.2977,0.5776]	[−3.8675,-2.0955]	[−0.1406,0.5243]
Residency area	0.4164	−3.0528^*^	0.1743
	(0.2304)	(1.4881)	(0.3431)
	[0.1408,1.2318]	[−5.9774,-0.1282]	[−0.5001,0.8486]
Childhood neighborhood relationship * residency area	1.2591	1.5145	0.2778
	(0.6914)	(1.4810)	(0.3533)
	[0.4292,3.6936]	[−1.3963,4.4253]	[−0.4167,0.9722]
**D. Education status difference**			
Childhood neighborhood relationship	1.0150	−2.2743	0.5076
	(0.4822)	(1.5626)	(0.4574)
	[0.4000,2.5757]	[−5.3454,0.7968]	[−0.3913,1.4065]
Education status	2.1107	0.1997	0.8621
	(1.0233)	(1.6108)	(0.4713)
	[0.8162,5.4587]	[−2.9662,3.3655]	[−0.0643,1.7884]
Childhood neighborhood relationship * education status	0.3806	−0.5687	−0.3126
	(0.1932)	(1.6491)	(0.4818)
	[0.1407,1.0295]	[−3.8098,2.6723]	[−1.2596,0.6343]
**E. Income difference**			
Childhood neighborhood relationship	0.4279^***^	−3.2355^***^	0.2913
	(0.0988)	(0.7296)	(0.2012)
	[0.2721,0.6729]	[−4.6694,-1.8015]	[−0.1042,0.6867]
Income	0.9913	−0.1131	0.0264
	(0.0372)	(0.1083)	(0.0311)
	[0.9210,1.0669]	[−0.3259,0.0997]	[−0.0348,0.0875]
Childhood neighborhood relationship * income	0.9989	0.1046	−0.0145
	(0.0383)	(0.1101)	(0.0319)
	[0.9265,1.0770]	[−0.1119,0.3211]	[−0.0773,0.0482]

Robust standard errors that clustered at the community level are reported in parentheses. 95% confidence intervals are reported in square brackets. ^***^p < 0.001, ^**^p < 0.01, ^*^p < 0.05. Control variables and province fixed effects were controlled

In addition, we obtain evidence indicating that the interaction terms between childhood neighborhood relationship and gender, residency area, education status, and income were statistically insignificant (*p* > 0.05), which suggests that the association between childhood neighborhood relationship and mental health did not differ as for the above-mentioned dimensions.

## Discussion

Employing the data which was obtained from the 2014 and 2015 waves of CHARLS dataset, we investigated the association between childhood neighborhood relationship and mental health among the middle-aged and older adults in China. We obtain robust evidence indicating that the middle-aged and older adults who lived in place where neighbors had close-knit relationships at childhood was significantly correlated with decreased odds of suffering from depressive symptoms in middle and later life. Furthermore, we also obtain robust evidence indicating that the middle-aged and older adults who lived in place where neighbors had close-knit relationships at childhood was significantly associated with a reduced CES-D score in middle and later life. The reason may lie in the fact that childhood circumstances have long-term effects on depression. These findings confirm the importance of living in place where neighbors had close-knit relationships at childhood. Some studies found that childhood adversity had a negative effect on depression in middle and later life [11, 13, 32]. Different from these studies, this study focused on the association between childhood neighborhood relationship and mental health among the middle-aged and older adults. At the same time, we also confirmed the long-term effect of childhood experience on depression. Moreover, it is found from the OLS regression models that childhood neighborhood relationship was not significantly linked to cognitive ability in middle and later life. In addition, it is important to notice that PSM and doubly robust estimation provide similar results.

The heterogeneity analysis indicates that childhood neighborhood relationship had a stronger association with the odds of suffering from depressive symptoms for the older adults who aged 60 years and older than for the middle-aged adults who aged 45–59 years. In addition, the heterogeneity analysis also indicates that childhood neighborhood relationship produced a stronger association with the CES–D score for the older adults who aged 60 years and older than for the middle-aged adults who aged 45–59 years. To some extents, these findings confirm the long-term effect of childhood neighborhood relationship on mental health. Furthermore, we obtain evidence indicating that the association between childhood neighborhood relationship and mental health did not differ by gender, residency area, education status, and income.

This study has two policy implications. On the one hand, it is necessary for Chinese people to pay more attention to the association between childhood neighborhood relationship and mental health and maintaining close-knit neighborhood relationships. On the other hand, the local governments need to strengthen the construction of community and organize some community activities to improve neighborhood social cohesion.

Some limitations for this study should be noted. Firstly, in CHARLS, the childhood neighborhood relationship was collected retrospectively, thus recall bias may exist in this study. Secondly, due to the cross-sectional design, we can not explore the causal relationship between childhood neighborhood relationship and mental health among the middle-aged and older adults. Future studies can consider using panel data to explore the causal relationship between childhood neighborhood relationship and mental health. Thirdly, due to the limitation of data, we can not investigate the association between the duration of living as a child in place where neighbors had close-knit relationships and mental health among the middle-aged and older adults. Last but not least, it is worth noting that we can not control some variables that may be associated with mental health, such as air pollution [33], due to the unavailability of data.

Despite these limitations, this study extends the literature by exploring the association between childhood neighborhood relationship and mental health among the middle-aged and older adults in China using a nationally representative dataset. In addition, this study also explores the heterogeneity of the association between childhood neighborhood relationship and mental health by incorporating the interaction terms into the regression models. Moreover, given the fact that this study employed the data which was obtained from a nationally representative survey, the results of this study can be generalized to the whole China. Methodologically, this study used the PSM and doubly robust estimation to conduct a robustness check, which may encourage the further applications of causal inference methods in health outcome issue.

## Conclusion

In conclusion, this study demonstrates that the middle-aged and older adults who lived in place where neighbors had close-knit relationships at childhood was significantly correlated with decreased odds of suffering from depressive symptoms. Furthermore, this study also demonstrates that the middle-aged and older adults who lived in place where neighbors had close-knit relationships at childhood was significantly associated with a reduced CES–D score. Moreover, it is found that childhood neighborhood relationship was not significantly linked to cognitive ability in middle and later life. The results were robust to a variety of specifications. In addition, childhood neighborhood relationship was significantly linked to greater decreases in the odds of suffering from depressive symptoms and CES–D score for the older adults who aged 60 years and older than for the middle-aged adults who aged 45–59 years. The integrated interventions, including maintaining close-knit neighborhood relationships and strengthening the construction of community, may be useful to improve mental health.

## Data Availability

The data used in this study were derived from China Health and Retirement Longitudinal Study (CHARLS) in the year of 2015. Available at: http://charls.pku.edu.cn/zh-CN.

## Abbreviations

CHARLS: China Health and Retirement Longitudinal Study
CES-D: Center of Epidemiological Survey-Depression Scale
VIF: Variance Inflation Factor
OLS: Ordinary Least Squares
PSM: Propensity Score Matching
AIPW: Augmented Inverse-probability Weighting
IPWRA: Inverse-probability-weighted Regression Adjustment.
